# Morbidities Worsening Index to Sleep in the Older Adults During COVID-19: Potential Moderators

**DOI:** 10.3389/fpsyg.2022.913644

**Published:** 2022-06-27

**Authors:** Katie Moraes de Almondes, Eleni de Araujo Sales Castro, Teresa Paiva

**Affiliations:** ^1^Department of Psychology and Postgraduate Program in Psychobiology, AMBSONO Sleep Clinic, Onofre Lopes University Hospital, Federal University of Rio Grande do Norte, Natal, Brazil; ^2^Postgraduate Program in Psychobiology, Federal University of Rio Grande do Norte, Natal, Brazil; ^3^CENC –Sleep Medicine Center, Lisbon, Portugal; ^4^ISAMB – Faculdade de Medicina, Universidade de Lisboa, Lisbon, Portugal; ^5^Comprehensive Health Research Center (CHRC), Universidade Nova de Lisboa, Lisbon, Portugal

**Keywords:** older people, sleep habits, sleep disorders, insomnia, COVID-19

## Abstract

Older adults were considered a vulnerable group for the COVID-19 infection and its consequences, including problems with sleep.

**Aim:**

To evaluate the prevalence of sleep disorders in older adults, to describe their sleep patterns, as well as to analyse if there were any changes in comparison with the period pre-pandemic.

**Materials and Methods:**

Online survey used for data collection received answers from 914 elderly age range 65–90 years, from April to August 2020. Results: 71% of the sample reported a pre-existent sleep disorder, and some of them worsened during the pandemic, especially Insomnia in women and Obstructive Sleep Apnea in men. No difference in sleep duration before and during the pandemic was found, although there was a worsening of some aspects related to sleep, such as sleep quality, sleep efficiency, awakening quality, sleep latency and nocturnal awakenings, especially in the female gender. Educational level influenced sleep latency, indicating higher sleep latency among those with primary education when compared with the ones with Ph.D.

**Conclusion:**

The pandemic had influenced sleep patterns among the elderly, as well as worsening of pre-existent sleep disorders. Female gender and low educational level were considered risk factors for sleep alterations, and high educational level, on its turn, appeared to be a protective factor.

## Introduction

The worldwide pandemic of COVID-19 caused by the severe acute respiratory syndrome coronavirus 2 (SARS-CoV-2) (Zhu et al., 2020) left consequences beyond the disease-specific respiratory symptoms. The clinical scenario can follow an asymptomatic course or range from milder symptoms such as dyspnoea to acute respiratory distress syndrome, which might lead to death (Zhou et al., 2020).

One of the outcomes was the imposed lockdown in several cities around the world, which has changed the routine of many people. Essential aspects such as behaviour, social interactions, emotion (Salehinejad et al., 2021) and sleep habits (Cellini et al., 2020) have been deeply affected. Worldwide, the emergence and deterioration of sleep disorders and the increase in sleep pattern changes have been observed in the general population (Gupta and Pandi-Perumal, 2020).

The vulnerability of older adults in pandemic situations is dependent on biology, behaviour, gender, social isolation, social assistance, and healthcare availability (Doraiswamy et al., 2020). With the COVID-19 outbreak, they were considered a vulnerable group to suffer severe forms of this infection (Brooke and Jackson, 2020; Cardinalli et al., 2020). This concern was primarily due to the association between old age and the decline in immune function that could cause higher hospitalisation and mortality (Karlberg et al., 2004). They also face a natural decrease in the size of the upper airway (Martin et al., 1997), as well as an age-dependent decrease in nasal resistance (Xu et al., 2019), which may represent risk factors.

Sleep-related factors have also been suggested as one explanation for the COVID-19 negative outcomes at older ages. Older adults face a natural deterioration of the normal sleep process, which is associated with negative health consequences (Neikrug and Ancoli-Israel, 2010). Furthermore, melatonin levels also naturally reduce with age, which can contribute to disrupting circadian rhythmicity (Cardinalli et al., 2020).

Thereby, the elders were encouraged, more than any other age group, to self-isolate at home as a way of protection. The effects of home confinement, though, represented a challenge to the maintenance of a regular sleep-wake schedule, contributing to a circadian misalignment (Pires et al., 2021). Social zeitgebers, such as outdoors activities or participation in religious or other social groups were dramatically reduced. Consequently, the natural light exposure was decreased, which is the main driver of human circadian rhythmicity and influences changes in sleep timing (Salehinejad et al., 2021).

Older adults were also considered a vulnerable group to experiencing psychological symptoms during this pandemic, affecting sleep quality. In this regard, literature shows mixed results, as some studies found a high percentage of psychological symptoms among them, such as depression and anxiety (Dziedzic et al., 2021; Maggi et al., 2022), while others reported low levels of psychological distress, probably due to resilience and coping strategies (Ouanes et al., 2021). What is crucial to understand is that the mental state of the elderly also influences their sleep quality and that these aspects must be considered.

Among older adults, sleep disorders and complaints commonly have a prevalence variation between 20 and 50% (Gulia and Kumar, 2018). Not surprisingly, sleep alterations have been a common complaint in this pandemic (Cellini et al., 2020) such as sleep quality (Trabelsi et al., 2021), awakening quality (Paiva et al., 2021), emergence and worsening of sleep problems (Barros et al., 2020), especially sleep disorders like insomnia (Morin et al., 2021).

A sizable part of the studies on sleep problems during the pandemic has shown that insomnia is the sleep disorder with the most prevalent alterations (Cénat et al., 2021), followed by Obstructive Sleep Apnea (Thorpy et al., 2020) and Restless Leg Syndrome (Franco et al., 2020). Sleep has restorative and regulatory properties (Mackiewicz et al., 2007) and helps to modulate the immune system (Salehinejad et al., 2021), so sleep problems can weaken immunity and increase organism susceptibility to infections (Ibarra-Coronado et al., 2015). It can also affect the regulation of emotional processing, making people more reactive to stressful events (de Almondes et al., 2021). Therefore, good sleep quality becomes notedly relevant to better cope with stressful situations.

Nevertheless, a plethora of researchers found that during this period, older adults had a less negative impact on sleep compared to other age groups (Barros et al., 2020; Bidzan-Bluma et al., 2020; Beck et al., 2021; Paiva et al., 2021). Some authors suggest that despite the impact elders had, they were less affected by the pandemic due to more resilience, not to mention that most of them did not have to worry about job loss (Taylor et al., 2008).

This variability can be due to several aspects, such as the age category among the elders, as the younger group among older adults (60–70 years old) seems to have had the greatest impact on sleep (Emerson, 2020). A review of sleep patterns and mood associations in older adults during the pandemic found many studies that reported important changes in sleep during this period, but also a few studies that found no changes in sleep in this population (Cipriani et al., 2021).

Some authors also placed the concept of sleep reactivity, which suggests that stress exposure disrupts sleep. Thus, individuals who are high-sleep reactive may develop sleep disturbances in response to a stressful situation (Drake et al., 2014; Gupta and Pandi-Perumal, 2020), whereas those with low sleep reactivity would not face sleep problems as a response to stress (Kalmbach et al., 2018). This can be an explanation for why some people have more sleep problems while others do not, regardless of the situation.

The study aimed to evaluate the prevalence of sleep disorders in older adults, describe their sleep patterns, as well as to analyse changes in comparison with the period pre-pandemic. We elaborated the Morbidity Worsening Index to Sleep to analyse the worsening of disorders and their relationship with psychosocial factors.

## Materials and Methods

### Setting and Study Population

The survey was developed for this research project, but it used as background knowledge the questionnaires routinely applied to the patients of CENC – Sleep Medicine Center and the literature concerning health and risk-related habits, sleep schedules and associated habits and comorbidities.

The survey was made online by the Survey Legend^®^ platform and it was targetted to also include the elderly, from 65 years old. Surveys were anonymous, allowing data analysis and statistical use and it was released during the 1st COVID-19 wave in Portugal, from April to August 2020. During this period, to control the spread of the virus, several restrictions on economic activity and social life were imposed, such as the cancellation of public events, school closures, and restriction of national and international movements, among many others (Peixoto et al., 2020; Coutinho, 2021).

The overall project was approved by CENC’s Ethical Committee 1/2020. The study was announced on the CENC’s website^1^, on the Facebook pages of the team participants, on the websites of the supporting professional associations (Medical, Nurses, Psychologists, and Pharmaceutics) and by the sleep laboratories involved in the study, in the site of the supporting professional associations and *via* letters to most of the trade unions. There was no funding, public or private and no conflict of interests.

A total of 914 elderly from 65 to 90 years old completed the questionnaires. Independent older adults, regardless of age, responded to the survey without support, and the ones who needed had support from a caregiver or family member. The study included data obtained in Portugal (Continent and Islands: Madeira and Azores). The inclusion criteria were survey answering. The exclusion criteria were not accepting statistical anonymous data use, incomplete surveys and inconsistent answers (i.e., being on sick leave at 90 years of age).

### Questionnaires

The first page of the survey included the research’s purpose, author identification, approval by the Ethical Committee, names of a contact person in case of need, and supporting entities.

The study had 68 questions, subdivided into topics, as follows:

1. Sociodemographic characteristics – questions about age, gender, civil status, and education level.
2. Characteristics of housing during confinement – questions about type of house, house location and how many people were living in the house.
3. COVID-19 infection in self and family – questions about infection, infection severity, and death of someone by COVID or other cause.
4. Sleep habits – questions about bedtime and time to get up (both on weekdays and on weekends), sleep duration (self-reported in hours), sleep latency (self-reported in minutes), awakenings (self-reported in number of awakenings during the night), sleep quality and sleep awakening quality (evaluated in a VAS 1–10, meaning the higher the score, the poorer the sleep/awakening quality) and sleep efficiency (self-reported sleep duration/sleep time *100, expressed as a percentage) pre and during COVID.
5. Sleep disorders – yes/no questions about existing sleep disorders, such as insomnia, sleep apnea, delayed sleep-wake phase disorder, restless leg syndrome, bruxism, hypersomnia, periodic limb movements disorder and shift work disorder. The same procedure was used to ask the participants which disorders worsened, which improved, and which did not change during COVID.

Participants were also asked about existing chronic diseases before COVID and if there was a worsening or no changes during the lockdown period. They were also asked about self-perceived psychological symptoms of depression, anxiety, irritability, worries and economic problems, for they may represent risk factors for the worsening of sleep disorders and sleep quality.

### Data Analysis

Qualitative variables were described by absolute (*n*) and relative frequencies (%), while quantitative variables were calculated by the mean and standard deviation. The normality of the data was tested by Kolmogorov-Smirnov test. Most continuous variables had a normal distribution. Comparisons of sleep disorders and worsening of sleep disorders, as well as of sleep disorders and gender were made using χ^2^-test; comparisons of worsening of sleep disorders and psychosocial factors were made using ANOVA; and *t*-test was used to compare sleep habits before and during COVID, as well as gender and sleep habits. The analysis was made in IBM SPSS Statistics 27.0.

## Results

### Sociodemographic Data

The survey was answered by 914 older adults aged > 65 years old. Overall, 59.3% of the sample were males and 40.7% were females. The average age of the elderly was 69.9 years. Most of the elderly were married (71.4%) and most of them had a graduate degree or higher (60.6%) and living with one more person as a companion (only 15.9% were alone) Age, civil status, educational level, and people living in the same house are presented in Table 1.

**TABLE 1:** Demographic characteristics of the participants (*n* = *914*).

Variables		Elderly > 65–90 years	
		*N*	%
Gender	Male	542	59.3%
	Female	372	40.7%
Civil status	Married	653	71.4%
	Bachelor	36	3.9%
	Widow	82	8.9%
	Divorced	113	12.3%
	Union	28	3.0%
Level of education	Primary	59	6.5%
	Secondary	169	18.5%
	Bachelor degree	71	7.9%
	Graduate	434	48.0%
	Master	70	7.7%
	Ph.D.	44	4.9%
Number of people in the house	One more	496	54.3%
	Two more	128	14%
	Three more	44	4.8%
	Four more	23	2.5%
	Five or more	33	3.6%

### COVID Infection

The great majority of participants in this study were not infected by COVID-19 (97.9%). Of the 2.1% infected, only 0.3% required hospitalisation. Regarding infection of friends and family, 10.6% of the sample reported knowing a friend or familiar who was infected by COVID-19 and among those, 0.4% died by the virus.

### Sleep Disorders

Considering sleep disorders, when asked about the presence of some sleep disorder pre-COVID-19, 71% of the elderly reported having at least one sleep disorder, the most prevalent being Obstructive Sleep Apnea, with more than 45% within the sample, followed by Insomnia, reported by 13.9% of them. The frequencies are presented in Table 2.

**TABLE 2:** Sleep disorders in the elderly pre COVID.

Sleep disorders pre-COVID	Disorder present	Disorder not present
	% (*N*)	% (*N*)
Insomnia	13.9% (124)	86.1% (765)
Sleep apnea	45.7% (406)	54.3% (483)
Bruxism	3.8% (34)	96.2% (855)
Hypersomnia	3.4% (30)	96.6% (859)
Delayed sleep-wake phase disorder	1.7% (15)	98.3% (874)
Sleep wake disorder	1.2% (11)	98.8% (878)
Parasomnia	0.1%(1)	99.9% (888)
REM sleep behavior disorder	1.2% (11)	98.8% (878)
		

Gender comparisons were also made considering the worsening of each pre-existent sleep disorder. As we can see in Table 3, some sleep disorders significantly worsened during the pandemic, specifically during the first lockdown, which was the period when participants answered the survey. The significant changes were in Insomnia and Restless Leg Syndrome in women and Sleep Apnea in men.

**TABLE 3:** Gender comparisons and sleep disorders worsening.

		No	Yes	χ^2^	*p*
Insomnia_worse	Male	362	39	11.966	0.001*
	Female	242	56		
Apnea_worse	Male	389	12	4.694	0.030*
	Female	296	2		
Restless Leg Syndrome_worse	Male	400	1	9.307	0.002*
	Female	289	9		
Hypersomnia_worse	Male	377	24	1.342	0.247
	Female	286	12		
Sleep Phase Delay_worse	Male	397	4	1.251	0.263
	Female	292	6		

*Statistically significant difference, p < 0.05.

In the Morbidities section, we found a variety of sleep disorders that the elderly had since before the pandemic. The Morbidity Index (MI) to sleep is the sum of all referred sleep morbidities at baseline with respect to COVID-19 in terms of worsening (Morbidities Worsening Index – MWI). In Table 4 we can see the effect of sleep morbidities upon global health status evaluated by the Morbidity Worsening Index to Sleep. It is distinct that some disorders had significant effects upon morbidities worsening, such as Insomnia, Sleep Apnea, Delayed Sleep –Wake phase disorder and Hypersomnia. To put it in other words, the elders who had one of these disorders had worse global health status.

**TABLE 4:** Sleep disorders and its worsening in the elderly during the pandemic.

Morbidity index	Elder > 65 years %(*n*)	χ^2^	*P*-value	χ^2^	*P*-value
				
				Morbidity worsening index	
Insomnia	13.9 (124)	15,115	0.00	51,566	0.000*
Sleep apnea	45.7 (406)	665,444	0.00	5,545	0.000*
PLMS or RLS	3.8 (34)	23,114	0.000	0,495	0.480
Delayed sleep-wake phase disorder	1.5 (15)	4,624	0.03	14,302	0.000*
Hypersomnia	3.3 (30)			11,074	0.001*
Parasomnia	0.1 (01)	2,865	0.091	1,515	0.218
REM behavior disorder	1.2 (11)	2,292	0.130	–	–

*Statistically significant difference, p < 0.05.

MWI was analysed with the psychosocial factors, as presented in Table 5. Worsening of insomnia, restless leg syndrome and hypersomnia were significantly correlated with depression (*Z* = 43.966, *p* < 0.001; *Z* = 10.993; *p* = 0.001; *Z* = 10.958; *p* = 0.001), anxiety (*Z* = 50.712, *p* < 0.001; *Z* = 34.227; *p* = 0.016; *Z* = 68.766; *p* = 0.001), irritability (*Z* = 47.778, *p* < 0.001; *Z* = 50.181; *p* = 0.003; *z* = 25.878; *p* < 0.001), and economic problems (*Z* = 13.107, *p* < 0.001; *Z* = 20.252; *p* < 0.001; *z* = 7.407; *p* = 0.007). Irritability and economic problems were also correlated with Sleep Apnea (*z* = 18.705, *p* < 0.001; *z* = 2.232, *p* = 0.015, respectively). These results suggest that the elderly who experienced a worsening of one of these sleep problems during the COVID pandemic also experienced worsening in the psychosocial aspects to which they were related. That indicates that psychosocial symptoms of depression, anxiety and irritability, as well as economic problems during this pandemic added to the worsening of sleep disorders.

**TABLE 5:** Morbidities worsening index and psychosocial factors in the elderly.

Morbidity worsening	χ^2^	*P*-value	Worsening	Depression				Anxiety				Irritability				Economic problems				Worries			
				*N*	Mean	*Z*	*p*	*N*	Mean	*Z*	*p*	*N*	Mean	*Z*	*p*	*N*	Mean	*Z*	*p*	*N*	Mean	*Z*	*p*
None worse	93.495	<*0*.*001*	No	433	3.42	1.542	0.215	431	3.91	0.513	0.474	431	3.78	2.464	0.117	434	2.93	2.045	0.153	431	5.64	1.204	0.273
			Yes	242	3.19			244	3.77			243	3.49			242	2.69			240	5.42		
Insomnia			No	582	3.11	43.966	<*0*.*001*	580	3.6	50.712	<*0*.*001*	581	3.43	47.778	<*0*.*001*	582	2.73	13.107	<*0*.*001*	577	5.38	21.144	<*0*.*001*
			Yes	93	4.75			95	5.45			93	5.2			94	3.57			94	6.67		
Apnea			No	661	3.32	1.477	0.225	661	3.84	3.133	0.077	660	3.62	18.705	<*0*.*001*	662	2.82	2.232	0.015	657	5.54	2.232	0.136
			Yes	14	4.07			14	5			14	6.36			14	4.21			14	6.57		
Restless legs syndrome			No	665	3.3	10.993	0.001	665	3.84	34.227	0.016	664	3.64	50.181	0.003	660	2.8	20.252	<*0*.*001*	661	5.54	3.886	0.055
			Yes	10	5.7			10	5.7			10	5.9			10	5.8			10	7.1		
Hypersomnia			No	639	3.27	10.958	0.001	640	3.79	68.766	0.001	639	3.57	25.878	<*0*.*001*	640	2.79	7.407	0.007	636	5.54	1.084	0.298
			Yes	36	4.56			35	5.23			35	5.63			36	3.78			35	6		

### Sleep Habits

In general, the elderly from our sample did not have to change their sleep duration during the pandemic. Nevertheless, some sleep parameters had changed between the pre and mid-pandemic periods, as can be seen in Table 6. There were changes regarding sleep latency, sleep quality, sleep awakening quality, awakenings, and sleep efficiency, suggesting an impact on their sleep caused by the imposed lockdown.

**TABLE 6:** Comparisons of sleep parameters before and during COVID.

	Mean		*t* (df)	*P*-value
	Before COVID	During COVID		
Sleep duration	6.94	7.03	−1.43 (677)	0.153
Time into bed	0.13	0.18	−1.162 (719)	0.246
Time out of bed	8.0	8.2	−4.41 (748)	<0.0001
Sleep latency	24.5	30.9	−7.34 (639)	<0.0001
Awakenings	2.2	2.5	−3.23 (551)	<0.0001
Sleep quality	6.5	6.1	8.84 (693)	<0.0001
Sleep awakening quality	6.8	6.4	7.46 (685)	<0.0001
Sleep efficiency	85.1	83.1	4.35 (602)	<0.0001

*df, degrees of freedom. T-test for all comparisons; Sleep duration: reported in hours; sleep latency: self-reported sleep latency in minutes; awakenings: self-reported number of awakenings during the night; sleep quality and sleep awakening quality: self-reported quality, with higher scores indicating better quality; sleep efficiency: self-reported sleep duration/sleep time × 100, expressed as a percentage.*

Sleep parameters were also analysed considering gender differences. Women exhibited higher sleep latency, higher frequency of awakenings during sleep, lower sleep efficiency, lower sleep quality and lower sleep awakening quality when compared to men (Table 7).

**TABLE 7:** Gender comparisons and sleep parameters.

	Mean		*t* (df)	*P*-value
	Male	Female		
Sleep duration	7.0	7.0	0.35 (685)	0.200
Sleep latency	23.9	41.8	−6.73 (643)	<0.0001
Awakenings	2.2	3.1	−2.97 (586)	<0.0001
Sleep Efficiency	84.5	80.7	2.94 (622)	<0.0001
Sleep quality	6.6	5.4	7.58 (693)	<0.0001
Sleep waking quality	6.8	5.9	5.90 (688)	<0.0001

*df, degrees of freedom. T-test for all comparisons; Sleep duration: reported in hours; sleep latency: self-reported sleep latency in minutes; awakenings: self-reported number of awakenings during the night; sleep quality and sleep awakening quality: self-reported quality, with higher scores indicating better quality; sleep efficiency: self-reported sleep duration/sleep time × 100, expressed as a percentage.*

Analyses were made to see if the educational level had an effect on sleep parameters during the pandemic, such as sleep duration, sleep latency, awakenings, sleep efficiency, sleep quality and sleep-waking quality. One-Way ANOVA indicated that there was an effect of educational level on sleep latency (*Z* = 2,611; *p* < 0.005). Bonferroni *post hoc* test showed that the group with primary education differed from the group with Ph.D. (*p* < 0.001). This result suggests that a high educational level may have acted as a protective factor for sleep latency. One-Way ANOVA was also used to analyse differences between the number of people living in the house and the same sleep parameters, but no differences were found between groups. This result suggests that having a companion in the house during the pandemic may not have influenced the sleep quality of the elders.

## Discussion

This is the first research, to our knowledge, to elaborate a Morbidity Worsening Index to Sleep for older adults, that could analyse the worsening of sleep morbidities pre-pandemic and during the pandemic of COVID-19. We hypothesised that the pandemic would have a negative impact on the older adults’ sleep, but it would be modulated by protective and risk factors.

As expected, we found a high prevalence of sleep disorders in our sample. Sleep Apnea was the most prevalent, followed by Insomnia. As our sample had more men than women, it may explain a higher percentage of Sleep Apnea, as this disorder is most prevalent in the male gender (Senaratna et al., 2017), while the female gender is more likely to develop insomnia when compared to men (Zhang and Wing, 2006; Zeng et al., 2020). Other disorders were not as prevalent, but we should consider the possibility that they were not previously diagnosed. It is important to distinguish, though, that some disorders cannot be directly associated with COVID-19. For instance, REM sleep behaviour disorder is more related to idiopathic or symptomatic of a neurologic disorder (Schenck and Mahowald, 2002), especially alpha-synucleopathies (Claassen et al., 2010).

Regarding sleep habits, women presented significantly more alterations during the pandemic when compared to men, including higher sleep latency, more awakening episodes, less sleep efficiency, less sleep quality, and less waking quality than men. These results suggest that women suffered worse negative consequences in sleep patterns during the pandemic when compared to men, as found in other studies (Bajaj et al., 2020; Franceschini et al., 2020; Beck et al., 2021).

Women generally appear to be more prone to certain problems, as several studies found a higher prevalence of health problems among them (Beck et al., 2021). Authors discuss that it might be related to the fact that women are more aware and observant about their symptoms and health problems (Zhang and Wing, 2006). When it comes to the period of the pandemic, one study reported a higher percentage of women who experienced worsening of a previous sleep problem when compared to men (Barros et al., 2020). It can also be related to the intensification of women’s routines with household chores, not to mention that many elderly women had to take care of their grandchildren for a longer period, as schools remained closed.

Another study, though, found worse sleep and psychological conditions in women at the beginning of the lockdown, but symptoms of insomnia, for instance, reduced over time while men reported worsening sleep quality and insomnia (Salfi et al., 2020). Our findings are in line with the literature, as in our study, following the prevalence of each disorder regarding sex, also the worsening of insomnia was significantly higher among women, but the worsening of Sleep Apnea was significantly higher among men.

Thereby, both genders suffered from a worsening of a previous sleep disorder during the beginning of the confinement. Notwithstanding the result of worsening Sleep Apnea in men, this disorder is more related to aspects such as age, male gender, and body mass index, among others (Franklin and Lindberg, 2015). Considering that Sleep Apnea was a pre-existent condition and not directly related to emotional state changes caused by the pandemic, we should consider the possibility of elders being left unattended during the first lockdown, which may have contributed to the worsening of this condition. However, we have no data to inform us if this was the case.

Regarding psychological symptoms related to COVID-19, literature also shows worse responses in the female gender (Fenollar-Cortés et al., 2021; Maggi et al., 2021, 2022; Yan et al., 2021), but similar to the study of Salfi and collaborators mentioned above, another study showed that the gender differences decreased significantly after some weeks of confinement, meaning that women learnt how to adapt and respond better to this unprecedented situation (Fenollar-Cortés et al., 2021).

It is important to understand that sleep parameters are also influenced by other factors, such as current personal worries (Bajaj et al., 2020). In our analysis of sleep disorders and psychosocial factors, we found an association between the worsening of some sleep disorders and symptoms of depression, anxiety and irritability among the elderly. Another study analysed that those who reported a decrease in perceived sleep quality due to COVID-19 reported significantly higher state anxiety, insomnia symptoms, and poorer sleep quality (Bigalke et al., 2020). These results might help to shed light on the relationship between psychosocial factors and sleep, shown in several studies both before (Fang et al., 2018) and during the pandemic (Barros et al., 2020; Deng et al., 2020).

Although there were negative impacts and changes on the elderly’s sleep during this pandemic, they continued to present relatively good sleep quality and awakening quality, as well as good sleep efficiency. Considering this, it is important to rise the existence of possible protective factors that may have helped them to cope with this pandemic.

Firstly, most of our sample were people with high educational levels, which is associated with higher income conditions (Bajaj et al., 2020). Those conditions were found to be linked with better quality of life (Bidzan-Bluma et al., 2020), better coping strategies (Chen, 2020), and protectors against worsening sleep problems (Bajaj et al., 2020; Barros et al., 2020).

Furthermore, the great majority of them were living with at least one more person in the house during the lockdown, which may have helped them reduce the feeling of loneliness. It is well known that having a companion during stressful situations is considered a protective factor against loneliness, especially among the elderly who are prone to social isolation even under normal circumstances (Philip and Cherian, 2020). In the present study, associations with the ones living alone and worsening of sleep were not found, possibly due to a small number of lonely participants, which is a limitation regarding data.

The feeling of loneliness caused by isolation may result in several health alterations, including in sleep (Grolli et al., 2021). In a study on loneliness and sleep problems, a higher level of COVID-19 related loneliness was associated with higher levels of sleep problems among older adults (Grossman et al., 2021).

Social isolation has also been one of the main concerns during this period among the elderly and may be one of the factors that contributed to emotional distress and feelings of loneliness (Pant and Subedi, 2020). Isolation has a negative impact on physical, mental and cognitive health (Novotney, 2019) and it is potentially caused by living alone (Cacioppo et al., 2011). Living alone is also associated with more susceptibility to changes in lifestyle and behaviours (Lehtisalo et al., 2021).

As most of our participants were living with someone else in the house, we believe that this was a protective factor against emotional distress and therefore, it contributed to certain stability in their sleep, despite the perceived changes.

Another aspect to consider is that just a small number of participants in our sample were infected by the virus, and this could have positively influenced their mental state. COVID-19 related worries were associated with sleep problems in the older age (Grossman et al., 2021), and great levels of depression, anxiety and sleep disturbances were found in COVID-19 patients (Deng et al., 2020). It is relevant to point out that when COVID-19 was declared a world pandemic, nobody knew the future outcomes or how long it would take until things were back to normal, causing great concern worldwide.

Notwithstanding these factors that may have protected the elderly from an even worsening in their sleep, their worsening in sleep disorders was significant and associated with worsening of psychosocial factors, such as depression, anxiety and irritability. These findings are in line with results from other studies conducted during this pandemic, in which these symptoms were prevailing (Bigalke et al., 2020; García-Fernández et al., 2020). In an international survey of the elderly launched also during the first lockdown, researchers found that the frequency of participants with high mental wellbeing decreased, whereas there was an increase in the frequency of participants with probable and possible depression or anxiety from pre- to during lockdown (Trabelsi et al., 2021).

This study had sundry limitations. As it was based on participants’ subjective self-report questionnaires, it is reasonable to expect a sampling bias. Also, given the dynamics of the virus and the extended period of quarantine in Portugal, it is important to point out that this data refers only to the period in which they were collected.

Also, this survey was only reached by the elderly who had access to the internet, a factor that may not be representative of the entire elderly population, especially the ones with lower income conditions and lower educational levels.

Psychiatric and neurological conditions also have an effect on sleep, but this could not be fully addressed in this study. Despite participants being asked about comorbidities, we believe that elderly with worse conditions either did not access the research or were not aware of their condition, not to mention elders with dementia, who were deeply affected by the quarantine due to the COVID-19 pandemic (Rainero et al., 2021). In contrast, this survey may have attracted older adults with more concern and symptoms of sleep problems, which may explain the relatively high prevalence of sleep disorders in the sample.

## Conclusion

The COVID-19 related-lockdown altered sleep habits and worsened sleep disorders among older adults. Although the changes in sleep habits were mild, alterations in sleep disorders were associated with symptoms of depression, anxiety and irritability among the elderly. Some factors may have acted as protective factors among participants, such as high level of education, not being alone during lockdown and not being infected by the coronavirus. However, as they are considered a vulnerable group for infection, public policies must also consider their psychological vulnerabilities for improving the well-being of this population during and after the pandemic. Moreover, as many elders have morbidities, the first step would be to ensure health care for this population so that their morbidities do not worsen during social isolation. Future studies should investigate the long-term effects on mental well-being and sleep in older adults.

## Data Availability Statement

The original contributions presented in this study are included in the article/supplementary material, further inquiries can be directed to the corresponding author.

## Ethics Statement

The studies involving human participants were reviewed and approved by CENC’S Ethical Committee. The patients/participants provided their written informed consent to participate in this study.

## Author Contributions

KA and TP: study design. KA, TP, and EC: writing the draft, integration of the authors’ comments, final manuscript, contributed to the article, and approved the submitted version.

## Conflict of Interest

The authors declare that the research was conducted in the absence of any commercial or financial relationships that could be construed as a potential conflict of interest.

## Publisher’s Note

All claims expressed in this article are solely those of the authors and do not necessarily represent those of their affiliated organizations, or those of the publisher, the editors and the reviewers. Any product that may be evaluated in this article, or claim that may be made by its manufacturer, is not guaranteed or endorsed by the publisher.

## Footnotes

1
www.isleep.pt
